# Temperature-sensitive mutations for live-attenuated Rift Valley fever vaccines: implications from other RNA viruses

**DOI:** 10.3389/fmicb.2015.00787

**Published:** 2015-08-11

**Authors:** Shoko Nishiyama, Tetsuro Ikegami

**Affiliations:** ^1^Department of Pathology, The University of Texas Medical Branch at Galveston, Galveston, TXUSA; ^2^Sealy Center for Vaccine Development, The University of Texas Medical Branch at Galveston, Galveston, TXUSA; ^3^Center for Biodefense and Emerging Infectious Diseases, The University of Texas Medical Branch at Galveston, Galveston, TXUSA

**Keywords:** Rift Valley fever virus, bunyavirus, vaccine, MP-12, temperature sensitivity

## Abstract

Rift Valley fever (RVF) is a mosquito-borne zoonotic disease endemic to the African continent. RVF is characterized by high rate of abortions in ruminants and hemorrhagic fever, encephalitis, or blindness in humans. RVF is caused by the Rift Valley fever virus (RVFV: genus *Phlebovirus*, family *Bunyaviridae*). Vaccination is the only known effective strategy to prevent the disease, but there are no licensed RVF vaccines available for humans. A live-attenuated vaccine candidate derived from the wild-type pathogenic Egyptian ZH548 strain, MP-12, has been conditionally licensed for veterinary use in the U.S. MP-12 displays a temperature-sensitive (ts) phenotype and does not replicate at 41°C. The ts mutation limits viral replication at a specific body temperature and may lead to an attenuation of the virus. Here we will review well-characterized ts mutations for RNA viruses, and further discuss the potential in designing novel live-attenuated vaccines for RVF.

## Life Cycle for RVFV

Rift Valley fever virus has a tripartite negative-stranded RNA genome designated Small (S)-, Medium (M)-, and Large (L)-segments. The S-segment encodes two open reading frames (ORF) for a nucleoprotein (N) and a non-structural protein (NSs) in an ambi-sense manner. The M-segment encodes a single ORF for a polyprotein precursor. The precursor protein is co-translationally cleaved into four different proteins: Gn, Gc, 78-kD protein, and a non-structural protein (NSm). The L-segment encodes a single ORF for the RNA-dependent RNA polymerase (L) protein.

DC-SIGN, dendritic cell specific intercellular adhesion molecule-3-grabbing non-integrin, is a receptor for RVFV and binds to oligosaccharides attached to virions (Lozach et al., 2011). After viral attachment, viral entry occurs via caveola-mediated endocytosis in a pH-dependent manner (Harmon et al., 2012). Upon uncoating, the L protein, derived from incoming virions begins viral mRNA synthesis (primary transcription). The viral polymerase cleaves a capped host mRNA, near the 5′ terminus, and uses it to prime the synthesis of viral mRNA (cap-snatching) (Schmaljohn and Nichol, 2007). As soon as viral proteins accumulate, the viral RNA genome becomes encapsidated with N protein and forms the ribonucleocapsid (RNP), which is used for RNA genome replication. The viral envelope proteins, Gn and Gc, play a role in viral assembly. Gn encodes a Golgi retention motif (Gerrard and Nichol, 2002), while Gc localizes to the ER, when Gn is not present. The complexes of Gn and Gc localize to the Golgi and trigger the assembly of RNP and L, and then the budding of virions (Piper et al., 2011).

Rift Valley fever virus encodes two non-structural proteins, NSs and NSm. Both proteins are dispensable for viral replication. However, NSs serves as a major virulence factor as it counteracts host antiviral responses. NSs suppresses host general transcription by interrupting the assembly of transcription factor (TF) IIH, which is essential for the function of cellular RNA polymerase I or II (Le May et al., 2004; Kalveram et al., 2011; Kainulainen et al., 2014). RVFV NSs also suppresses the up-regulation of interferon (IFN)-β promoter at a transcriptional level by interacting with cellular transcription repressors (Billecocq et al., 2004; Le May et al., 2008). Furthermore, RVFV NSs promotes the degradation of dsRNA-dependent protein kinase (PKR). PKR is a cellular sensor of dsRNA or the 5′-triphosphate of ssRNA. Upon the binding to RNA, PKR is dimerized. PKR homodimers then undergo autophosphorylation and phosphorylate eukaryotic initiation factor (eIF) 2α, which inhibits the initiation of cellular and viral translation. By promoting the degradation of PKR, RVFV can synthesize viral proteins without inducing significant eIF2α phosphorylation (Habjan et al., 2009; Ikegami et al., 2009). The minor virulence factor, NSm, inhibits the apoptosis of infected cells, yet the lack of NSm expression only moderately affects the RVFV mortality in mice (Won et al., 2006; Terasaki et al., 2013; Kreher et al., 2014). The 78-kD protein and NSm contribute to an efficient dissemination of RVFV in mosquitoes (Crabtree et al., 2012; Kading et al., 2014; Kreher et al., 2014).

## RVFV ts Mutants

Rift Valley fever virus is an arbovirus and can replicate in both mosquito and mammalian hosts in nature. RVFV can replicate at 28°C in insect cells (Weingartl et al., 2014), and at 41°C in mammalian cells (Saluzzo and Smith, 1990). Internal body temperatures of RVFV-susceptible hosts are as follows: sheep: 38.3–39.9°C, cattle: 38.0–39.3°C, goats: 38.5–39.7°C, humans: 37°C, mice: 37.5–38.0°C (Talan, 1984; Robertshaw, 2004). RVFV replication initially occurs in the draining lymph nodes, liver, and spleen (Smith et al., 2010; Gommet et al., 2011). It is important to understand the “restrictive temperature” for the ts mutants, because it can allow prediction of viral replication at specific body temperatures in mammalian hosts.

**FIGURE 1:**
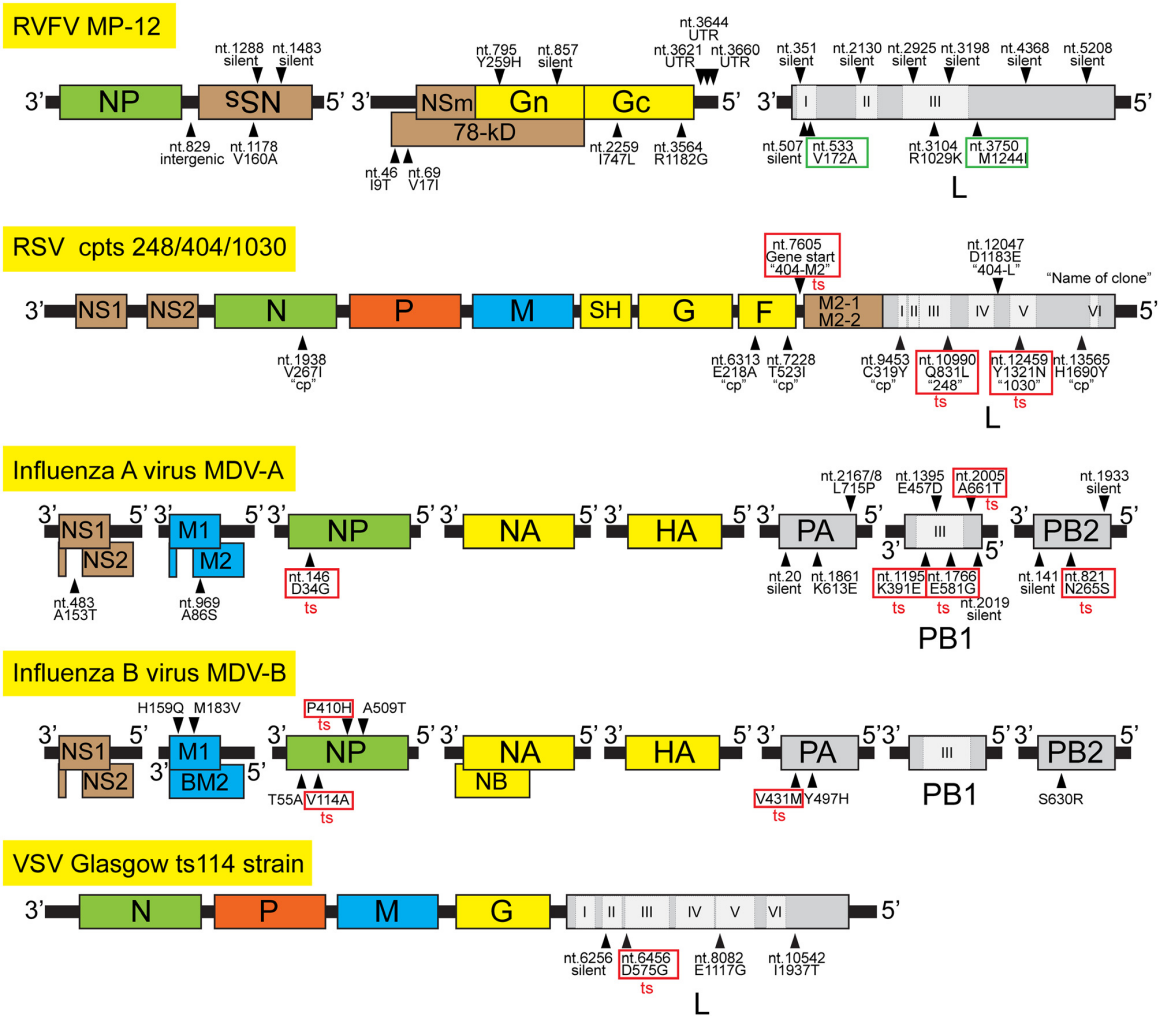
**Mapping of temperature-sensitive (ts) mutations for selected negative-stranded RNA viruses.** The genome structures of RVFV MP-12 strain, Respiratory syncytial virus (RSV) cpts 248/404/1030, Influenza A virus (MDV-A), Influenza B virus (MDV-B), and Vesicular stomatitis virus (VSV) ts114 strain are shown. Viral genes are shown in different colors: Nucleocapsid protein (green), phosphoprotein (orange), RNA-dependent RNA polymerase (gray), envelope protein (yellow), matrix proteins (blue), and accessory or non-structural proteins (brown). Conserved six functional regions of RNA-dependent RNA polymerase (RdRp) among non-segmented negative-stranded RNA viruses are shown as I, II, III, IV, V, and VI, and those aligned to RdRp of bunyavirus and influenza virus are also indicated. Location of representative mutations and ts mutations (red square) are indicated by arrowheads. Putative ts mutations in the L-segment of MP-12 are shown in green squares. For RSV mutations, the name of clone, which encodes the mutation, is also shown in quotation.

Currently, little is known about ts mutations for RVF vaccine candidates. The RVFV MP-12 strain was developed by 12 serial plaque isolations in human lung diploid (MRC-5) cells in the presence of a chemical mutagen, 5-fluorouracil (Caplen et al., 1985). As a result, a total of 23 mutations are encoded in the genome: four mutations in the S-segment, nine mutations in the M-segment, and 10 mutations in the L-segment (**Figure 1**). The MP-12 vaccine does not replicate efficiently *in vivo*, though the S-segment encodes a functional NSs gene. Saluzzo and Smith (1990) previously characterized reassortant RVFV strains between the pathogenic Senegal ArD38661 strain and the MP-12 vaccine strain or the intermediate passage levels of MP-12 (MP-4, MP-6, or MP-9). Their study identified that MP-12 M- and L-segment produce the ts phenotype. Ts mutations on the M-segment were introduced during the MP-12 development (from 7 to 9 passages). On the other hand, ts mutation on the L-segment occurred during the earlier stages of development (the passage 4 or earlier). Since the U533C (V172A) and G3750A (M1244I) mutations were introduced in the L-segment at the passage 3, these two specific mutations may be responsible for the ts phenotype of L-segment (Vialat et al., 1997). However, no further characterization of ts mutations has been reported for MP-12 vaccine.

Currently, the MP-12 vaccine is conditionally licensed in the U.S., and the master seed is available for the production of vaccine lots. A number of safety and efficacy tests were performed for the MP-12 vaccine using pregnant and newborn ruminants (Morrill et al., 1987, 1991, 1997a,b, 2013a; Morrill and Peters, 2003, 2011a,b). To understand the mechanism of attenuation for the MP-12 vaccine, virulent recombinant ZH501 (rZH501) strains encoding the MP-12 S-, M-, or L-segment, or a single mutation of the MP-12 M- or L-segment were analyzed in an outbred CD1 mouse model (1 × 10^3^ pfu, i.p) (Ikegami et al., 2015). The study revealed that an incorporation of a MP-12 S-, M-, or L-segment confers partial attenuation to pathogenic ZH501. Two amino acid changes in Gn (Y259H) and Gc (R1182G) were identified as major attenuation mutations for the M-segment. A combination of Y259H and R1182G only partially attenuates rZH501, while a combination of Y259H, R1182G, plus an L-segment mutation, G3104A (R1029K), could fully attenuate rZH501. Importantly, MP-12 encoding reversion mutations in these three amino acids (H259Y, G1182R, and K1029R) still retained attenuation in mice, indicating that the attenuation of MP-12 vaccine is supported by multiple attenuation mutations, and MP-12 does not revert into virulent phenotype by a few reversion mutations. Further characterization of ts mutations of MP-12 vaccine will help the understanding of the mechanism behind attenuation.

Meanwhile, Rossi and Turell (1988) isolated another ts strain of RVFV. RVFV T1 strain was isolated from female *Culex pipiens*, which fed on hamsters infected with the pathogenic ZH501 strain. T1 strain displayed a ts phenotype at 41°C, and produced uniformly small plaques. The T1 strain is also highly attenuated in hamsters, and the LD_50_ is >6.3 × 10^5^ pfu (i.p). On the other hand, the RVFV T46 strain, which was isolated from *Aedes taeniorhynchus* that fed on ZH501-infected gerbils, also predominantly produced small plaques, but was pathogenic in hamsters, without showing a ts phenotype. As the full genome sequences are available (T1 strain: GenBank Accession DQ375407, DQ380201, and DQ380150, T46 strain: DQ375405, DQ380147, and DQ380199), we analyzed the mutations that occurred in the T1 and T46 strains compared to the parental ZH501 strain. The T1 strain encodes two mutations in the N gene: the G144U (G to V) mutation and a deletion of A at nt.640, which causes a frame-shift and a premature termination of N protein synthesis. T1 strain also encodes a mutation in the 5′-M-untranslated region (C3818U), and two silent mutations in the L-segment (C282U and A2691G). On the other hand, T46 strain encodes only one mutation in the M-segment (U1174A: M to K). The T1 strain has not been further evaluated for vaccine development.

## Studies of ts Phenotype in Other RNA Viruses

It is difficult to predict viral attenuation, as a result of mutagenesis, without using animal models. However, a ts phenotype indicates attenuation *in vivo* and can be screened for using culture cells. Ts phenotypes have been characterized for many viruses (Richman and Murphy, 1979), using different approaches. In **Table 1**, we have summarized the ts mutants of selected RNA viruses. The ts phenotype depends on host cell types for the Poliovirus Sabin Type2 strain or Dengue virus NS5 gene mutants. For other RNA viruses, the majority of ts mutants were determined by using just one or a few cell types. Thus, it is important to broadly test different cell types to determine a ts phenotype. The location of ts mutations results in a unique ts phenotype. If the viral polymerase encodes a ts mutation, the syntheses of viral genomic RNA or mRNA, or both can be affected at a restricted temperature. If envelope proteins encode a ts mutation, the production of infectious progeny can be inefficient at a restricted temperature. A lack of viral replication or viral RNA synthesis can be an indicator for ts screening when a ts mutation is encoded in the viral polymerase. On the other hand, when the ts mutation is encoded in envelope proteins, the reduction of viral titers may be more remarkable than the decrease in viral RNA accumulation.

**Table 1:** Determination of temperature-sensitive (ts) phenotypes for RNA viruses.

Classification	Species	ts strains	Location of ts mutation	Restrictive Temp for ts (Permissive)	Cell type	Reference
**Family *Bunyaviridae***						
Genus *Phlebovirus*	RVFV	MP-12	M- and L-segments	41°C (35°C)	Vero	Rossi and Turell (1988)
		T1	Unknown	41°C (35°C)	Vero	Rossi and Turell (1988)
	UUKV	S23 ts6, 7, 8, 11, 12	Unknown	39°C (33°C)	CEF	Gahmberg (1984)
Genus *Orthobunyavirus*	MAGV	MAG ts8	M-segment	38°C (33°C)	BHK-21	Pollitt et al. (2006)
	LACV	RFC/25B.5	Unknown	39.8°C (37°C)	BHK-21	Endres et al. (1990)
	BUNV	rBUNdelNSs with N mutation	N protein	38°C (33°C)	VeroE6	Eifan and Elliott (2009)
	SSHV	ts1, 2, 3	Unknown	39.5°C (33°C)	BHK-21	Gentsch and Bishop (1976)
	AKV	OBE-1 strain mutants	M- and/or L-segments	40°C (33°C)	HmLu-1	Ogawa et al. (2007)
**Family *Paramixoviridae***						
Genus *Pneumovirus*	RSV	rA2 cpts 248/404	M2 and L	37°C (32°C)	Hep-2	Whitehead et al. (1998)
		rA2 cpts 248/404/1030	M2 and L	36°C (32°C)	Hep-2	Whitehead et al. (1998)
Genus *Respirovirus*	HPIV3	rHPIV3 JS cp45	L	38°C (32°C)	LLC-MK2	Skiadopoulos et al. (1998)
	HPIV1	rHPIV1 L:F456L	L	38°C (32°C)	LLC-MK2	Newman et al. (2004)
	BPIV3	rBPIV L:I1103V	L	40°C (37°C)	Vero	Haller et al. (2001)
**Family *Orthomyxoviridae***						
Genus *Influenza virus A*	Flu A	MDV-A	NP, PB1, PB2	39°C (33°C)	MDCK	Jin et al. (2003)
Genus *Influenza virus B*	Flu B	MDV-B	M1, NP, PA	37°C (33°C)	MDCK or PCK	Hoffmann et al. (2005)
**Family *Rhabdoviridae***						
Genus *Vesiculovirus*	VSV	Indiana ts11, 13, 114, 22, 33, 41, 45	L (ts114)	38.5°C (31°C)	L	Rettenmier et al. (1975)
**Family *Picornaviridae***						
Genus *Enterovirus*	PV	Sabin Type3	VP3	40°C (35°C)	Hep-2c	Minor et al. (1989)
		Sabin Type2	5′-UTR	39.9°C (35°C)	Hep-2c	Macadam et al. (1991)
			5′-UTR	38.4°C (35°C)	BGM	
			5′-UTR	38.3°C (35°C)	Vero F	
		Sabin Type1	VP1, VP3, VP4, 3D^pol^, 3′-UTR	40°C (37°C)	HeLa S3	Bouchard et al. (1995)
	EV A	EV71 (BrCr-ts)	VP1	39°C (36°C)	Vero	Arita et al. (2005)
**Family *Flaviviridae***						
Genus *Flavivirus*	DENV	rDEN4 NS5 E645A-K646A	NS5	39°C (35°C)	Vero	Hanley et al. (2002)
				Not ts	HuH-7	
	WNV	rWNV NS4B C102S	NS4B	41°C (37°C)	Vero	Wicker et al. (2006)
	JEV	M1/311 ts104	Unknown	39°C (35°C)	CF	Halle and Zebovitz (1977)
	LGV	E5-104	NS3, E	37°C (32°C)	Vero	Rumyantsev et al. (2006)

*RVFV, Rift Valley fever virus; UUKV, Uukuniemi virus; MAGV, Maguari virus; LACV, La Crosse virus; BUNV, Bunyamwera virus; SSHV, Snowshow hare virus; AKV, Akabane virus; RSV, Respiratory syncytial virus; HPIV, Human parainfluenza virus; VSV, Vesicular stomatitis virus; PV, Poliovirus; EV, Enterovirus; DENV, Dengue virus; WNV, West Nile virus; JEV, Japanese encephalitis virus; LGV, Langat virus.*

## RNA-Dependent RNA Polymerase and ts Phenotypes

Temperature-sensitive mutations have been identified in the RNA-dependent RNA polymerases of many RNA viruses (**Table 1**). Non-segmented negative-stranded RNA viruses encode six conserved regions (Region I, II, III, IV, V, and VI) in the RNA-dependent RNA polymerase (Rahmeh et al., 2010). The region III (Premotif A, and Motif A, B, C, D, and E) serves in RNA polymerization, and V and VI function in cap addition and cap methylation, respectively. There is also an endonuclease domain at the N-terminus of some of segmented negative-stranded RNA viruses (Reguera et al., 2010). As described above, V172A and M1244I mutations may be involved in the ts phenotype for MP-12 L protein. The V172A mutation is located in Region I, while M1244I is located downstream of Region III Motif E (Muller et al., 1994). Though no studies have been performed for the ts phenotype of RVFV L mutants, mutagenesis of the L protein may identify ts mutations useful for the future rational design of RVF vaccines. Several studies have indicated that ts phenotypes occur from amino acid change(s) in the viral polymerase. **Figure 1** illustrates the locations of ts mutations for selected negative-stranded RNA viruses.

The vesicular stomatitis virus (VSV) ts114 mutant encodes three amino changes (D575G, E1117G, and I1937T) in the L-segment compared to the non-ts parental strain. The ts phenotype occurs from D575G, which is located between PreMotif A and Motif A in Region III (Galloway and Wertz, 2009). The ts114 mutant displayed a ts phenotype at 39°C. The ts114 mutant also showed a selected inhibition of viral mRNA synthesis, while maintaining active viral RNA genome replication. However, the selected inhibition of viral transcription only occurred with the combination of all three mutations of ts114, and the single D575G mutant abolished both viral genome replication and transcription at 39°C.

The respiratory syncytial virus (RSV) cold-adapted, temperature-sensitive (cpts) 248/404/1030 is a live-attenuated vaccine strain (Polack and Karron, 2004). It encodes two ts mutations (Q831L and Y1321N) in the L region, in addition to a nucleotide substitution in the M2 transcription start sequence. Q831L is located between Motif C and D in Region III, while Y1321N is located in Region V. In another study, an alanine scan of charged amino acid residues in the RSV L protein was performed to identify ts mutants (Tang et al., 2002). Alanine scanning identified three types of L phenotypes: (1) Abolished L activity, (2) Little change in L activities, and (3) a ts phenotype at 39°C: K157A-D158A (Upstream of Region I), E510A-R511A, R520A, L587A-R588A, R588A-D589A (Region II and upstream) or E1208A-R1209A (Region V).

The live-attenuated FluMist vaccine consists of master donor virus for influenza virus A (MDV-A) and influenza virus B (MDV-B). MDV-A has been developed by serial passages of the wt A/Ann Arbor/6/60 strain in primary chicken kidney tissue culture at successively low temperatures down to 25°C (Jin et al., 2003). MDV-A encodes five ts mutations in NP, PB1, and PB2. A study showed that the MDV-A virus has an impaired synthesis of anti-viral-sense genomic RNA, but not mRNA, at 39°C (Chan et al., 2008). MDV-A also decreases the nuclear export of RNP and the incorporation of the M1 protein into virions at 39°C. Furthermore, MDV-A virions become heterogeneous in size and shape at 39°C. Meanwhile, MDV-B is derived from a cold-adapted B/Ann Arbor/a/66 strain, and encodes ts mutations in the PA and NP segments (Hoffmann et al., 2005).

Temperature-sensitive mutants have been successfully developed as licensed vaccines, or candidate vaccines, in particular, for respiratory diseases: e.g., FluMist (influenza A and B viruses), FluAvert (equine influenza virus) (Paillot et al., 2006), and MEDI-559 (recombinant human RSV A2 cp248/404/1030/ΔSH) (Empey et al., 2010). Most ts mutants have been identified by random mutagenesis. Rational design of ts mutations by reverse genetics will require further understanding of temperature-susceptible domains.

## Efficacy of MP-12 Vaccine against Aerosol Challenge of Pathogenic RVFV

A vaccine protection from an exposure via aerosols or powders must be considered in the case of bioterrorism. Little is known about the efficacy of RVF vaccines against aerosol challenge of pathogenic RVFV. Efficacy of the MP-12 vaccine in a pathogenic RVFV challenge via the respiratory route has been studied. Aerosol (~1 × 10^5^ pfu) or intranasal vaccination (~1 × 10^4^ pfu in 1.0 ml volume) of rhesus macaques with MP-12 induced serum neutralizing IgG (Morrill and Peters, 2011a). Intramuscular vaccination of rhesus macaques with MP-12 vaccine also led to neutralizing antibody titers of 1:320 to 1:1,280 (Plaque Reduction Neutralization Test 80: PRNT_80_), which was maintained for 6 years. The vaccinated rhesus macaques were protected from an aerosol challenge of the pathogenic ZH501 strain (Morrill and Peters, 2011b). These results clearly indicate that the MP-12 vaccine is efficacious for aerosol RVFV challenge, regardless of vaccination routes. Meanwhile, further characterization of viral replications in upper and lower respiratory tract will be important to evaluate the risk of available live-attenuated RVF vaccine candidates: e.g., MP-12, or rMP12-ΔNSm21/384 (Morrill et al., 2013a,b), Clone 13 vaccine (Dungu et al., 2010), or rZH501ΔNSsΔNSm (Bird et al., 2011).

## Concluding Remarks

Outbreak of RVF causes decreased animal productivities and viral persistence in mosquito vectors for unknown periods of time, thus significantly impacting the animal industry. In the U.S., the live-attenuated MP-12 vaccine is conditionally licensed, but the vaccine will still require an improvement in terms of safety considering reported side effects: e.g., abortions in pregnant ewes, necrosis in calf liver. Further studies should design additional attenuation mutations rationally, including gene deletion(s) or ts mutations, to fully attenuate the S-, M-, and L-segments, toward the development of highly safe and efficacious RVF vaccines (Grobbelaar et al., 2011; Ikegami, 2012; Lihoradova and Ikegami, 2012).

## Conflict of Interest Statement

The authors declare that the research was conducted in the absence of any commercial or financial relationships that could be construed as a potential conflict of interest.
